# PortWeather: A Lightweight Onboard Solution for Real-Time Weather Prediction

**DOI:** 10.3390/s20113181

**Published:** 2020-06-03

**Authors:** Petros Karvelis, Daniele Mazzei, Matteo Biviano, Chrysostomos Stylios

**Affiliations:** 1 Department of Informatics and Telecommunication, University of Ioannina, 45110 Ioannina, Greece; stylios@uoi.gr; 2 Department of Computer Science, University of Pisa, 56126 Pisa, Italy; mazzei@di.unipi.it (D.M.); m.biviano@studenti.unipi.it (M.B.); 3 Computer Technology & Press “Diophantus”, 26504 Patras, Greece

**Keywords:** weather forecasting, microcontroller, IoT, maritime traffic

## Abstract

Maritime journeys significantly depend on weather conditions, and so meteorology has always had a key role in maritime businesses. Nowadays, the new era of innovative machine learning approaches along with the availability of a wide range of sensors and microcontrollers creates increasing perspectives for providing on-board reliable short-range forecasting of main meteorological variables. The main goal of this study is to propose a lightweight on-board solution for real-time weather prediction. The system is composed of a commercial weather station integrated with an industrial IOT-edge data processing module that computes the wind direction and speed forecasts without the need of an Internet connection. A regression machine learning algorithm was chosen so as to require the smallest amount of resources (memory, CPU) and be able to run in a microcontroller. The algorithm has been designed and coded following specific conditions and specifications. The system has been tested on real weather data gathered from static weather stations and onboard during a test trip. The efficiency of the system has been proven through various error metrics.

## 1. Introduction

Meteorology is the study of weather events, having a significant focus on the forecasting of principal weather variables. Prediction on weather conditions is performed by using historical data or mathematical models [1] or a combination of both. There is great importance in quantifying meteorological variables such as temperature, humidity, air pressure, and wind flow; their variations and interactions and how they change over time. For this reason, various spatial scales have been proposed in order to describe and predict weather conditions on local, regional, or global levels [2,3].

Weather conditions have had a significant effect on sea-transportation and all the maritime affairs over time [4]. Maritime activities are related to maritime commerce and maritime leisure, but they also affect the environment and consequently atmosphere and so weather conditions. All are related and interconnected and seem to belong to a vicious circle that impact the quality of the environment and the maritime meteorological conditions [5,6]. The safety of maritime transportation is strictly related to weather information. International Maritime Organization (IMO) [7] and the World Meteorological Organization (WMO) [8] have proposed and defined regulation strategies on how to provide marine weather predictions to minimize accidents and economic losses.

For these reasons, as also reported in [4], there is an essential need for maritime weather prediction systems, which is connected to the sustainable development of sea commerce. The development of advanced, real-time and onboard forecast systems is becoming of significant importance in order to achieve high weather forecast quality (especially with regard to storms, heavy precipitation, wind, waves, and extreme temperatures).

In the era of Machine Learning (ML), there are available several methods that could be investigated, adapted, expanded and tested in order to be used for weather prediction. The main approaches for weather prediction could be classified in the following four categories [9]:

- **nowcast:** which predicts the current wind speed;
- **short-term:** where the weather parameters are predicted for the next 72 h [10];
- **medium-range:** where the time scale increases to a period of 3–7 days and finally;
- **long-term:** where the weather conditions are predicted for a period from one week to months or even years [11].

When it comes to forecasting meteorological parameters like wind speed and wind direction, special care must be taken in order to tackle the problem of the local environment and the complexity of these parameters [12,13] which can seriously affect the performance of the forecasting methods [14,15]. Hence, it is a common approach to test several machine learning techniques to solve the weather forecasting problem [16,17]. However, all these algorithms can be classified into two main categories: online and trained. Trained models are ML algorithms that require a data-heavy learning phase before being able to produce predictions. These models are typically well-performing, but their forecasts are strongly linked to the training-set, thus making them not usable in moving scenarios like onboard weather prediction. On the contrary, online learning models are algorithms that continuously learn and adapt their predictions capabilities to the flow of data. These algorithms are the best candidates for the development of an onboard weather prediction system for maritime applications because the vessel position change does not influence them.

Nowadays, the Internet of Things (IoT) initiative has allowed communication and interoperability between machines [18,19,20] and so, in the specific application area, meteorological data collection has become more accessible and performant [21,22,23]. The IoT is, in fact, one of the disruptive and essential technologies for fields such as meteorology, since it allows data from different machines to converge on servers through the use of various technologies such as wireless sensor networks (WSN) and Low Power Wide Area Network (LPWAN) [24]. Moreover, the increase in the computational capabilities of IoT devices enabled the local processing of data pushing the IoT toward the “edge” paradigm [25,26]. In the edge paradigm, sensors are also endowed with local processing capabilities, thus making them able to provide real-time analysis. In these scenarios, the connection with remote servers is used only for data storage and synchronization, making network stability and availability not necessary for the functioning of the system.

In the meteorological field, it is possible through the IoT-edge paradigm to develop low-cost solutions [27] both for the collection and display of data and for the forecasting based on machine learning algorithms executed directly on the IoT nodes. The junction of IoT and Artificial Intelligence has been named AIoT and is considered one of the most promising technologies for the development of innovative systems and services of the Industry 4.0 era [28].

The goal of this work is to present a complete system named PortWeather, which forecasts the wind speed and direction at different horizons 30 min, 60 min, 90 min and two hours. The system consists of a commercial weather station which sends the current weather parameters to a local IOT unit based on a 32-bit micro-controller where the heart of the weather prediction algorithm runs without the need for a connection with a remote server.

The PortWeather system uses an online-learning ML weather forecast algorithm that does not require pre-train and the vessel position changes do not influence it. The system uses only local data to continuously train the weather predictor that produces the 30, 60, 90, and 120 min forecasts. Among various online learning algorithms developed for the weather forecast, a linear regression ML model has been chosen because it allows a lightweight implementation executable on a tiny microcontroller while still guaranteeing results comparable with more complex ML models.

Such integrated A-IoT edge solution has been designed for use on small professional and private boats that do not have the apparatus required for continuous connection to weather forecast remote services.

The PortWeather system has been tested on different datasets, including static data collected by ground weather stations and on a real sea trip where the system was installed on a test boat.

The PortWeather system can be easily installed on any typology of boats without requiring any training or ML/AI configuration. This feature makes the system easy to install, scalable, and also usable in long trips where the weather condition and micro-climate can change widely.

The manuscript is structured as follows: After the Introduction, Section 2 presents the state of the art in weather forecast systems and in ML algorithms currently used for weather prediction. Section 3 describes the PortWeather system, while Section 4 describes the preparation of the testing dataset and the results achieved by the forecast algorithm. Finally, Section 5 concludes the paper with some final remarks, recommendations, and suggestions for future work.

## 2. Related Works

### 2.1. Embedded Weather Forecasting Systems

Various weather station with forecasting capabilities are nowadays available on the market or have been developed as research activities. In [29], an Arduino based weather station is presented. The system collects data from various weather sensors processing them using a neural network. The network produces a “dressing index” that suggests to the users what would be the outdoor perceived temperature.

In [30,31], the creation of an embedded system for short-term weather forecast is presented. Like in the case of PortWeather, this system uses a tiny (PIC16F877) microcontroller as a computational unit. In this case, the forecast of meteorological variables (wind speed and direction, temperature, and relative humidity) is based on the juxtaposition of the data using five methods: Persistence Method, Trends Method, Climatology Method, Analog Method, and Numerical Weather Prediction Method. In this work, no machine learning method has been used, and no comparison to real data has been shown, making the system difficult to be compared with PortWeather or any other similar solution.

In [32], a low-power distributed IoT system has been developed for the prediction fo crop frost. The system is based on a network of weather parameters sensors connected through a Low Power Wide Area Network (LPWAN). In this work, all the sensors stream data to a central server that integrates them with data provided by public weather forecast services providing farmers and index of frost risk. Also in this case, the nodes use a microcontroller board, but no computation and processing of data are performed on-edge.

In [33], an interesting approach aimed at integrating Deep Learning (DL) weather forecast algorithms with IoT is presented. The system is composed of a series of data gathering node connected to a central server where the DL algorithm runs.

Most of the IOT and A-IOT (AI + IOT) systems for weather forecast have been designed as networks of sensors connected to a central server where all the AI/ML algorithms are executed. Moreover, most of these systems are based on ML algorithms that require data-intensive and difficult training phase.

To our knowledge, a system able to provide real-time local weather forecast using training-less ML algorithms executed on low-power, low-cost IOT units without the need of a continuous internet connection is still missing.

### 2.2. Machine Learning for Weather Prediction

A number of Machine Learning models have been already employed for the forecasting of different weather parameters. State-of-the-art techniques implemented successfully in this area include Artificial Neural Networks [34], Deep Learning techniques [35], Recursive Neural Networks [36], and Support Vector Machines [37]. On the other hand, there is a growing trend of combining various models or even ensemble methods [38].

All of these methods focus on batch learning where a ’batch’ of training data is provided, and the goal is to train the model that yields minimum error on predicting values. A significant disadvantage of these methods and their corresponding application is that they require big training dataset and enough memory to host them.

In [39], a performance analysis of time series forecasting models for short term wind speed prediction is presented. In this work, authors used a wind measurement station to collect: wind speed and direction, temperatures, humidity, and barometric pressure sensors. Six forecast algorithms have been tested: Random walk, Linear trend, Quadratic trend, Simple moving average, autoregressive moving average (ARIMA), and Nonlinear Autoregressive with an External Input (NARX) neural network model. The paper reports that the ARIMA and NARX models appear more successful than others demonstrating that regressive methods can be used for short time wind speed prediction.

In [40], another comparison of various algorithms for short-term weather prediction is reported. Authors highlight that online learning models such as Exponentially Weighted Moving Average (EWMA) and Simple Prior Moving Average (SPMA) perform better than static pre-trained neural networks while also allowing for avoiding the need of a training dataset for the priming of the model.

Indeed, there are specific types of machine learning processes where the model updates its parameters in a sequence of consecutive rounds. In these methods, there is no separation between the training phase and the testing phase, but the goal is to update the prediction model at the end of each trial to minimize the total loss over a sequence of trials [41]. Such type of learning process is called online learning [42] and arise in a variety of real-world applications, including prediction problems (e.g., forecasting the weather the next day) and decision/allocation problems (e.g., investing in different stocks or mutual funds) [43].

All these works highlight that online learning based prediction models are performing well in weather time series analysis and forecasting. Considering that they do not require a training phase and that are typically based on linear calculus, these models are good candidates for the development of server-free weather forecast IOT solutions.

In particular, linear regression models can be considered as an evolution of the Moving Average algorithm that allow a different weight for each parameter instead of the equal weight of each parameter in the case of the MA method. Moreover, Linear Regression allows a simple and lightweight implementation. For this reason, the PortWeather system prediction algorithm has been based on a linear regression model

## 3. The PortWeather System

Considering the complexity of performing weather forecasting on a boat without requiring an internet connection and algorithm pre-training, we decided to build a novel A-IOT system for sensor-driven real-time onboard weather prediction. The system is composed of: the weather station, which carries out continuous measurements of weather parameter and it is installed on the boat cabin roof; the data acquisition and elaboration unit and the machine learning algorithm that runs on the microcontroller of the data acquisition unit providing real-time weather predictions.

### 3.1. Hardware

**Figure 1:**
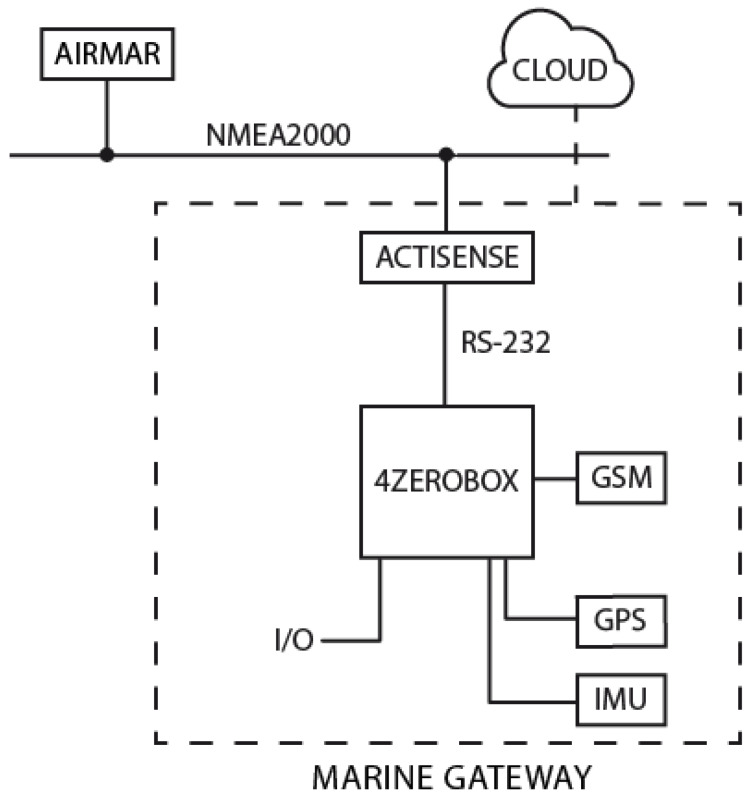
PortWeather system hardware diagram.

The hardware involved in the construction of the system consists of two main parts: a weather station and the data acquisition and elaboration unit (called “Marine Gateway”). An architectural diagram of the PortWeather system is illustrated in Figure 1.

#### 3.1.1. Weather Station

We installed a weather station (Airmar 220WX, Airmar Technology) on a tugboat unit. The Airmar station collects wind apparent speed and direction and, thanks to the availability of an integrated GPS and compass system, also calculates the real speed and direction of wind compensating the boat movement. Moreover, the data obtained from the GPS module are also used by the system for data and events chrono-referencing.

The weather station sends the data to the Marine Gateway using the NMEA 2000 protocol [44]. NMEA2000 is a widely used software protocol for maritime electronics that uses the CAN (Controller Area Network) as a transport protocol.

Table 1 reports technical details and features of the Airmar 220 weather station.

The weather station allows us to acquire a considerable amount of meteorological information in real time, through an all-in-one sensor system. Table 2 presents the data that the weather station gathers and provides:

#### 3.1.2. Marine Gateway

The Marine Gateway is the acquisition and elaboration unit of the PortWeather system. The unit acquires data from the weather station via NMEA2000 and elaborates them using the integrated regression algorithm. All the computation, analysis, and forecasting is performed locally on the Marine Gateway using the integrated 32-bit microcontroller. If a GSM internet connection is available, data are also sent to the cloud [45], allowing further data analysis and visualization.

In order to interface the Marine Gateway with the Airmar weather station, an Actisense NMEA2000 to RS-232 protocol converter has been used.

**Figure 2:**
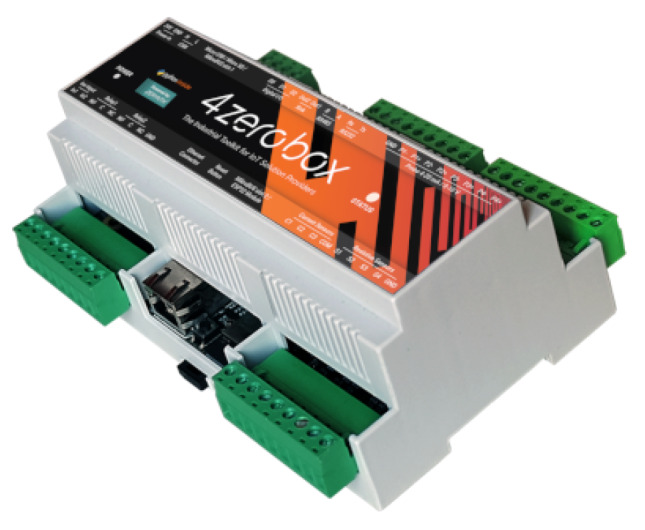
The 4ZeroBox device.

The Marine Gateway core is an industrial-grade development board called 4ZeroBox. The 4ZeroBox (displayed in the Figure 2) is a modular hardware electronic unit that simplifies the development of Industrial IoT applications [19] allowing rapid and seamless interfacing with industrial sensors, actuators, and Cloud services [46].

The 4ZeroBox computational unit is based on the powerful 32-bit ESP32 microcontroller made by Espressif Systems (Shangai, China) (240 MHz, 4 Mb Flash, 512 KB SRAM). The 4zerobox is programmable in Python (or hybrid C/Python) thanks to the Zerynth software (v. 2.5.2, Zerynth, Pisa, Italy) [20,47].

The 4zerobox allows acquiring data from both digital and analog ports, thus making it possible to interface with digital connection enabled machines and apparatus (like in the case of the Airmar station) but also with auxiliary analog external sensors [24,48].

The 4ZeroBox supports Wi-fi, Bluetooth, Ethernet, LoRa (a modulation technique that guarantees long-range communications), RS485, and RS232 connection while also integrating an SD Card for local data storage. Moreover, the system also integrates two onboard MikroBUS sockets to extend the 4ZeroBox with hundreds of other sensors available as MikroElektronika click boards.

In the PortWeather configuration, the two MikroBus expansion sockets have been used for the GSM 3G modem and an IMU (Inertial Measurement Unit) sensor.

When the GSM connection is available, data stored temporarily on the SD card are transferred to the Zerynth Device [49] services using the MQTT (Message Queuing Telemetry Transport) protocol.

Table 3 presents the technical description of the 4ZeroBox.

Technical specification of the ESP32 microcontroller at the core of the 4ZeroBox is reported in Table 4.

### 3.2. Software

In the designing and the development of the PortWeather system, the problems of high overhead, low efficiency, and low speed were addressed. A small memory footprint Machine Learning algorithm was selected, developed, and implemented using a regression method allowing the execution on a low memory microcontroller.

#### 3.2.1. Zerynth Platform

There are various hardware abstraction layer libraries, RTOSs, and interpreted language engines for the programming of microcontroller available on the market. However, considering the need for developing a Machine Learning model for weather prediction, we preferred to focus toward a complete and reliable developing framework able to support Python and, in particular, hybrid C/Python coding. For this reason, Zerynth [47] has been selected as the developing framework for the PortWeather project.

Zerynth [20] is a software suite composed of several tools that allow programming of 32-bit microcontrollers in Python or hybrid C/Python by also allowing a seamless connection of the implemented devices to various cloud platforms. Zerynth includes a compiler, debugger, and an editor, alongside various tutorials and example projects for a smooth learning experience. Moreover, Zerynth is natively supported by the 4ZeroBox, together with all various libraries necessary for the management of integrated peripherals, protocols, and sensors.

#### 3.2.2. Regression Methods

Regression is a type of problem that aims at predicting an output from a number of inputs using a limited number of data. Here, we use the following notation, $x\in R^{m}$, which describes the input features, and $y_{i}$ denotes the target/output value of a parameter like the wind speed (or the wind direction) that we need to forecast. Thus, $\left\{x_{i},y_{i}\right\}$ in our case will be training data and the list of training data is often called dataset. The dataset consists of a number *n* of training data $\left\{x_{i},y_{i}|i=1,...,n\right\}$. $R^{n\cdot m}$ denotes the input space, and $R^{n}$ the output space of *n* values. Regression is our basic problem since the target parameters $y_{i}$ (wind speed and direction) are continuous.

Thus, the whole problem is simply reduced to find a function like $X^{n\cdot m}\to Y^{n\cdot 1}$, for which we minimize the error between the real wind speed value and the predicted wind speed value. In this study, the available features are Temperature, Humidity, Wind direction and speed, and Barometric Pressure, while the value to predict are the wind speed direction. We also keep a record of the previous four values of each parameter which are fed as input to the regression model. One of the simpler algorithms that can be used for this purpose is Linear Regression (LR), which, in our case, seeks to predict the wind speed and direction as a linear combination of the input features.

In order to approximate *y* (wind speed and direction) as a linear functions of $x_{i}$:

(1) $$h_{\theta }\left(x\right)=\theta_{0}+\theta_{1}x_{1}+...+\theta_{m}x_{m}=\sum_{i=1}^{m}\theta_{i}x_{i}$$

where $x_{0}=1$ and *$\theta =\{\theta_{i}|i=1,...,m\}$* are the parameters of the linear function mapping from $X^{n\cdot m}$ to $Y^{n\cdot 1}$.

The common error function used to calculate the difference between the output $h_{\theta }\left(x\right)$ and the actual value $y_{i}$ is the least square error function defined in terms of:

(2) $$J\left(\theta \right)=\frac{1}{2}\sum_{i=1}^{n}{\left(h_{\theta }\left(x_{i}\right)-y_{i}\right)}^{2}$$

For the minimization of the error J(θ), a successful approach would be the Gradient Descent method which changes the values of the parameters repeatedly until convergence to a minimum value. The Gradient Descent method can be described as the following update on the parameters θ:

(3) $$\theta_{j}^{new}=\theta_{j}^{old}-a\frac{\partial J(\theta )}{\partial \theta_{j}}$$

where *a* is the learning rate that is usually set to $10^{-3}\leq a\leq 10^{-2}$ and smaller values for the learning rate leads to slower convergence.

However, in the case of microcontrollers, the Central Processing Unit (CPU) clock frequency is very low, and the memory is minimal. Thus, the machine-learning algorithm must be designed in order to tackle both of these problems. On the other hand, Stochastic Gradient Descent (SGD) [50] is an iterative procedure which has successfully used to optimize a function. The main advance of this SGD learning algorithm is that the parameters of the model are updated as the new data arrive [51]. This kind of procedure is currently gaining interest in the Deep Learning community [52].

In the case of the SGD algorithm, the update on the parameters is performed for a pair $\left(x_{i}.y_{i}\right)$ from the training set as:

(4) $$\theta_{j}^{new}=\theta_{j}^{old}-a\frac{\partial J(\theta ;x_{i}.y_{i})}{\partial \theta_{j}}$$

In SGD, the learning rate *a* is typically much smaller than any corresponding learning rate in Batch Gradient Descent algorithm because there is much more variance in the update.

In this work, the SGD algorithm was implemented through the use of a Flash Matrix table in which the acquired measurements are stored. The matrix is filled in a round-robin way so that those older measurements are eliminated in favor of more recent measurements.

The system has no pre-trained model saved. When the system boots, the first data are used for the learning of the algorithm; thus, the predictor does not produce any output. The method has only a short-term training memory, so it continuously adapts to current inputs making the system usable in any weather condition and geographical location. If the system is shut down for an extended period or if the sensors are changed, the system is thus able to adapt to the new conditions without requiring re-training.

However, all these conditions are infrequent in the maritime context where boats are used in limited geographical areas, and equipment change is due only to maintenance interventions.

Finally, the use of a low-power microcontroller-based data acquisition unit allows leaving the system always on also when the boat is harboured. This allows the PortWeather system to learn continuously being always ready for use.

## 4. PortWeather System Performance Tests

In order to test the prediction algorithm developed (both for the prediction of wind speed and wind direction), two main types of tests were performed: a static test and a dynamic test. In both cases, the results obtained for the predictions at 30, 60, 90, and 120 min were analyzed by calculating Median Absolute Error (*MAE*) and the Root Mean Square Error (*RMSE*), which are common measures for time series regression problems [53,54] and the mean absolute percentage error (*MAPE*) [55].

The measures are defined as:

(5) $$MAE=\frac{1}{N}\sum_{i=1}^{N}\left|{\hat{y}}_{i}-y_{i}\right|$$

(6) $$RMSE=\sqrt{\frac{1}{N}\sum_{i=1}^{N}{\left({\hat{y}}_{i}-y_{i}\right)}^{2}}$$

(7) $$MAPE=\frac{1}{N}\sum_{i=1}^{N}|\frac{{\hat{y}}_{i}-y_{i}}{y_{i}}|$$

where *N* is the number of the testing samples, ${\hat{y}}_{i}$ is the predicted value, and $y_{i}$ the true wind speed (or direction) value. However, *RMSE* may not be a good indicator of the average performance of the model [56]. Therefore, more importance will be given to the results obtained by the *MAE*.

We also calculated residual errors in the results of the predictions. The calculation of residual errors is realized as the difference between the predicted value ${\hat{y}}_{i}$ and the true wind speed (or direction) value $y_{i}$, and it is carried out in order to have further confirmation of the validity of the model.

### 4.1. Land Weather Station Test

The static test was carried out for the preliminary checks of the algorithm developed. In this case, the data on which the algorithm was executed was acquired by static/land weather stations. The data were collected from 6 November 2019 00:00:00 to 10 November 2019 13:45:00 from the weather stations of Livorno and Pianosa, [57].

**Figure 3:**
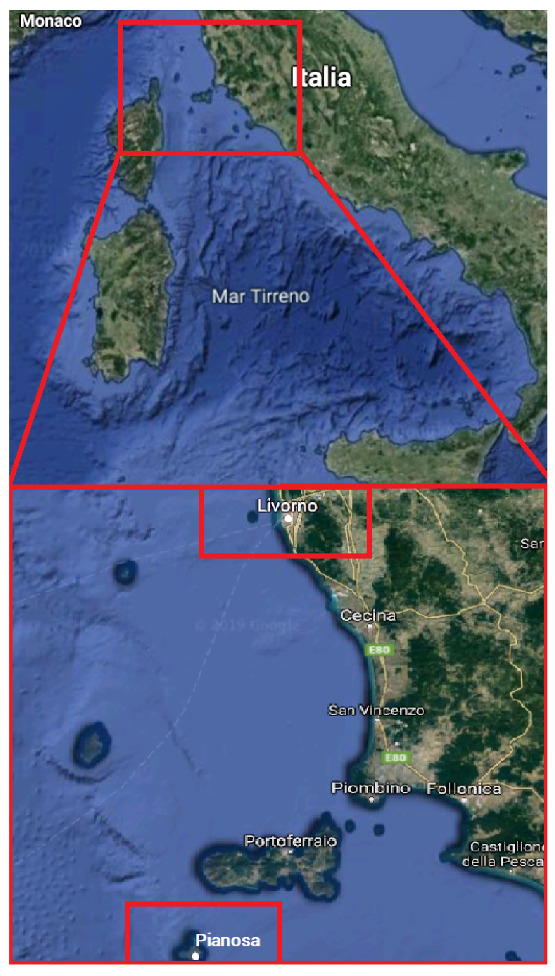
Lease of the Livorno and Pianosa weather stations.

Figure 3 displays the locations of the weather stations:

Table 5 and Table 6 provide a brief description of the data acquired from the land weather stations of Livorno and Pianosa, respectively.

**Figure 4:**
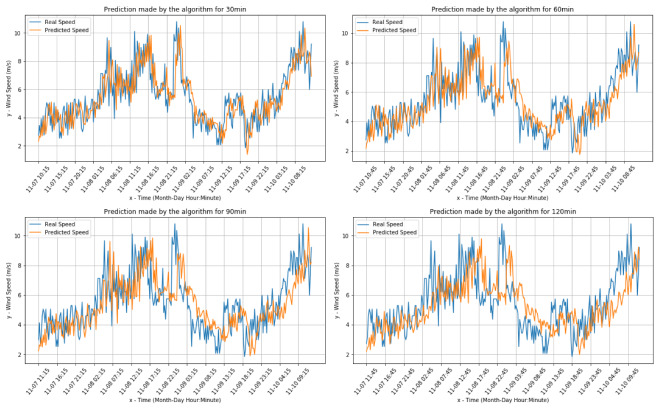
Wind speed predictions for Livorno data.

**Figure 5:**
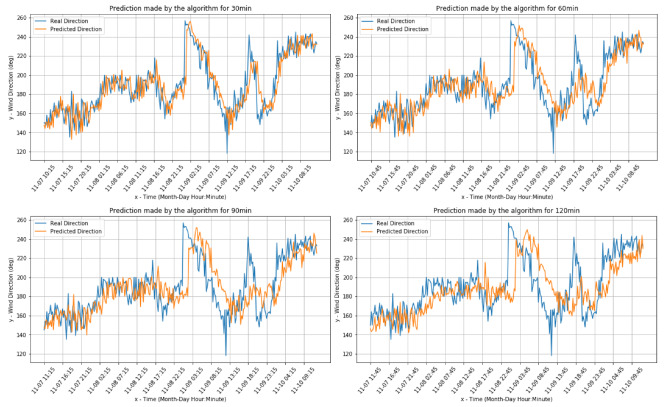
Wind direction real and predicted value for the Livorno weather station dataset.

Figure 4 and Figure 5 present the comparisons between the real values and the respective forecasts for the Livorno weather station dataset (both for wind speed and direction).

Table 7 presents the main results showing the wind speed error rates for the Livorno weather station. In particular, in this case, it is noted that the prediction of the wind speed realized by the algorithm differs by at most ±1.3 m/s, in the case of forecasts at two hours, with respect to the real speed.

The calculated errors have been gathered, record by record, and Table 8 presents the distribution of the residual values for the Livorno Weather station data set. It is concluded that the results obtained are consistent with what is reported in the calculation of the MAE, in fact, in the worst case, which is the 2-h prediction, most residual values belong to the range [−2, 2].

In the case of Livorno’s wind direction prediction, the algorithms differs by at most ±17.91°, in the case of forecasts at 2 h, as described in the following Table 9.

Table 10 presents calculation of the errors for the wind direction for the Livorno station, with the distribution of the residual values. It is inferred that the results obtained are consistent with what is reported in the calculation of the MAE, in fact, in the worst case, the 2-h prediction, most residual values belong to the range [−18; +18].

**Figure 6:**
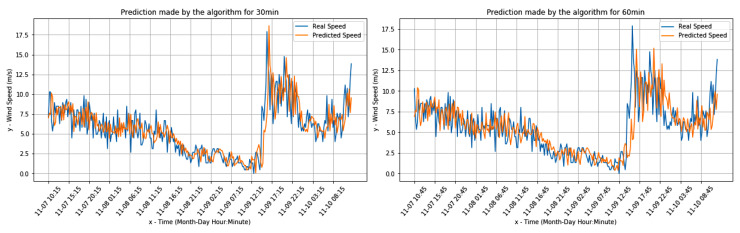

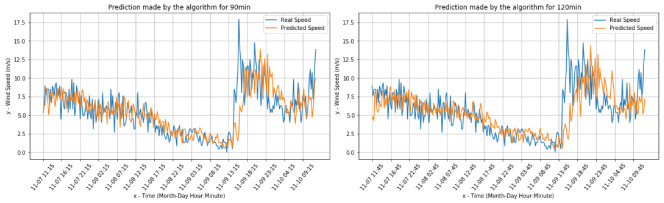
Wind speed real value and predictions for the Pianosa weather station data set.

**Figure 7:**
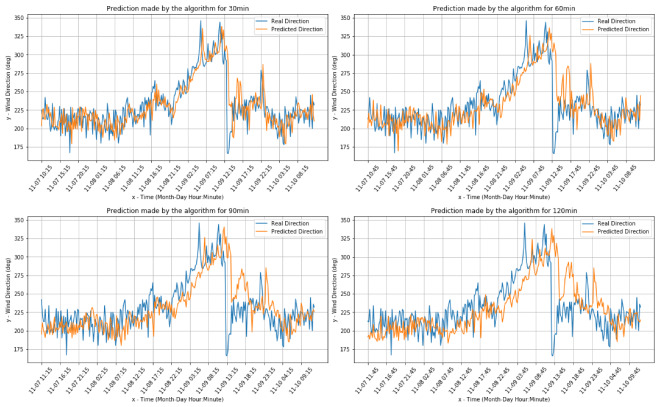
Wind direction real value and predictions for the the Pianosa weather station data set.

Figure 6 and Figure 7 present the comparisons between the real values and the respective forecasts for the Pianosa weather station dataset (correspondingly for wind speed and direction).

Table 11 presents the error rates for wind speed prediction for the Pianosa weather station. In this case, the prediction of the wind speed realized by the algorithm differs by at most ±1.7 m/s, in the case of forecasts at 2 h, with respect to the real wind speed.

The calculated errors have been gathered recorded by record, and Table 12 presents the distribution of the residual values for the Pianosa weather station. It is conducted that the results obtained are consistent with what is reported in the calculation of the MAE, in fact, in the worst case, the 2-h prediction, most residual values belong to the range [−2; +2]:

The worse prediction of the wind direction realized by the algorithm differs from the real direction by at most ±22.75${}^{\circ }$, in the case of forecasts at two hours for the Pianosa weather station as one can see in Table 13.

Table 14 presents the error distribution for the wind direction parameter for the Pianosa weather station. It can be observed that the results obtained are consistent with what is reported in the calculation of the MAE, in fact, in the worst case, the 2-h prediction, most residual values belong to the range [−25; +25].

Both from the analysis of the errors and the study of the graphs, it is concluded that the implemented algorithm reaches a high accuracy in predicting the wind speed and direction using statically collected data.

### 4.2. Dynamic Test

**Figure 8:**
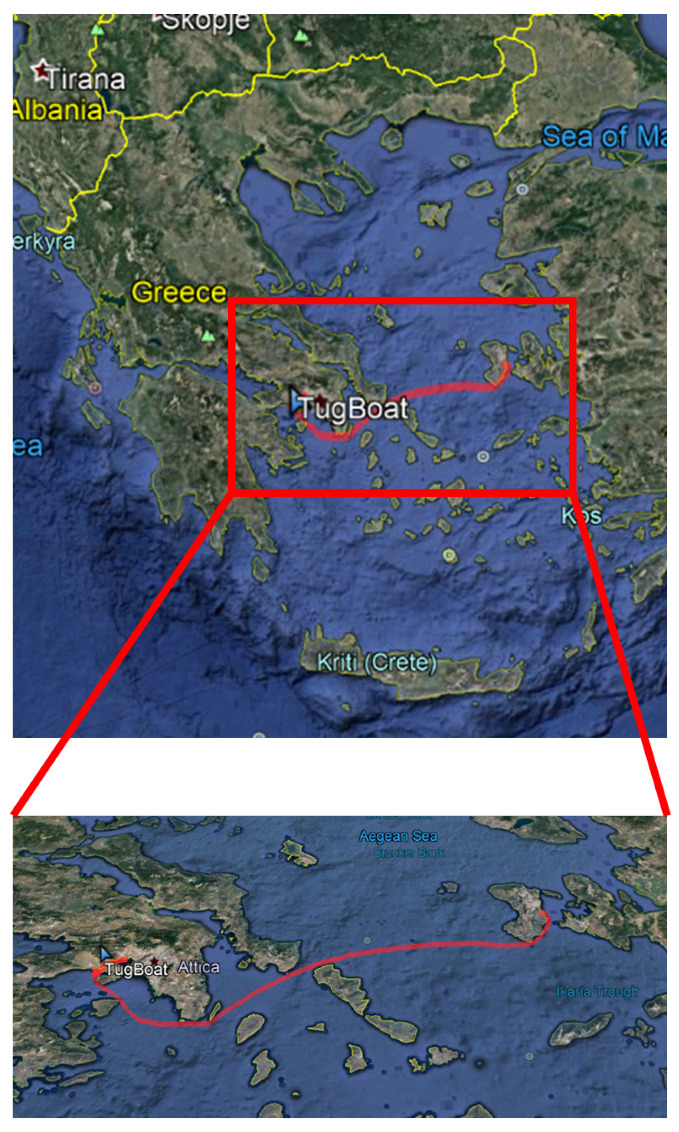
The recorded trip of the tugboat from Chios Island, Greece to Athens, Greece.

After evaluating the accuracy of the prediction algorithm on statically collected data, we tested the algorithm on dynamic data acquired using the PortWeather system installed on a tugboat. For the test, the tugboat made a trip from Chios island (Greece) to Athens (Greece), and the data collection lasted from 22 January 2018 11:24:00 to 9 February 2018 12:50:00. Figure 8 displays the planned trip of the ship.

Table 15 provides a brief description of the data acquired from the on-board weather station.

**Figure 9:**
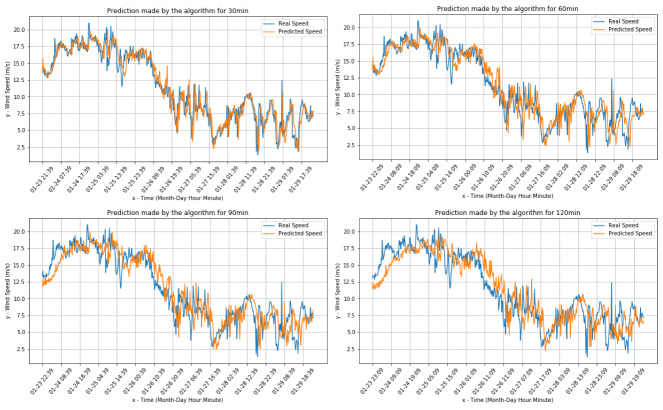
Real Wind speed and predictions for the onboard dataset.

**Figure 10:**
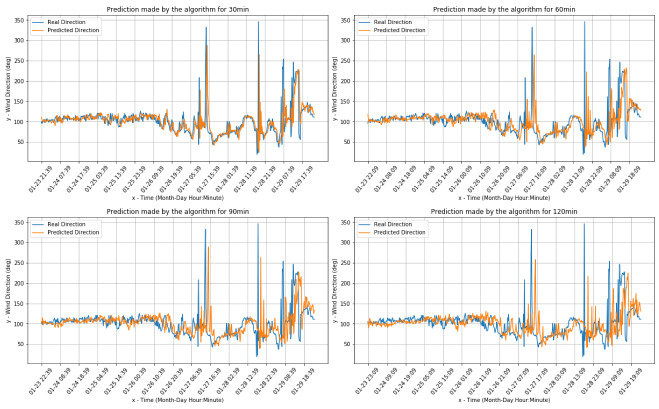
Wind direction predictions for the onboard dataset.

Figure 9 and Figure 10 present the comparisons between the real values and the respective forecasts (correspondingly for the wind speed and direction) for the onboard data set.

Table 16 presents the results showing the error rates for the onboard weather station. It is noted that the prediction of the wind speed realized by the algorithm differs by at most ±1.9 m/s, in the case of forecasts for 2-h prediction, with respect to the real wind speed.

The calculated errors have been recorded record by record, and Table 17 describes the distribution of the residual values. It is seen that the results obtained are consistent with what is reported in the calculation of the MAE, in fact, in the worst case, which is the 2-h prediction, most residual values belong to the range [−2; +2].

The worse prediction of the wind direction realized by the algorithm differs by at most ±20.3${}^{\circ }$, in the case of forecasts at 2 h, with respect to the real Wind direction as it can be seen in Table 18.

From the calculation of the errors, record by record, we have obtained Table 19 that describes the distribution of the residual values. The results obtained are consistent with what is reported in the calculation of the MAE, in fact, in the worst case, the 2-h prediction, most residual values belong to the range [−21; +21]:

### 4.3. Overall Evaluation

Table 20 and Table 21 present the MAE, MAPE, and RMSE error measures for the three different weather stations (Livorno, Pianosa and onboard) for the wind speed and wind direction, respectively. It can be inferred that, in terms of the MAE measure, the best accuracy of our method is performed for the Livorno data set for the wind speed and wind direction parameter. An increase of all the error measures is clearly reported with the increase of the prediction interval from 30 to 120 min.

The comparison of real and predicted value shows a time lag effect on the predicted data. As reported in [58], this effect is typical of simplified models where the prediction is highly correlated with a linear combination of historical data. However, although this effect is present, its visual analysis could lead to a misleading interpretation of the algorithm performances. Indeed, error measures analysis shows that the PortWeather system has relative low MAE, MAPE, and RMSE suggesting that the algorithm has an accuracy that is perfectly compatible with the kind of uses expected for the system.

## 5. Conclusions

This study presents an onboard weather prediction IOT system that provides short-term forecasts of wind speed and direction, without the need of an internet connection. The PortWeather system is composed of a commercial weather station interfaced with an Industrial IOT acquisition unit. The system is designed to be easy to install, reliable, and low-power, allowing the use on little and medium-sized boats. A forecasting algorithm based on linear regression was designed and implemented in order to work on a resource-constrained microcontroller for IOT applications. The implemented algorithm uses an online learning strategy allowing the system to work without the need of a training phase and Internet connection. This training-less approach allows the system to be resilient to boat geographical location shift and to recover from prolonged shutdown or sensors replacement quickly.

The efficiency of the system has been proven by testing the system in real sea conditions where weather parameters have been recorded on a tugboat operating in Greece. The algorithm performances have also been evaluated using other datasets, acquired from land weather stations located in different geographical and micro-climatic areas (Livorno and Pianosa, Italy).

As a general evaluation of the system, it is essential to highlight that linear regression algorithms are straightforward methods for the forecast of a time series evolution. These model are very simple, and sometimes it is possible to guess the output of these algorithms by looking at the time series plots. However, this is true for scientists but not for fishers and sea operators.

Maritime traffic security for little and medium-sized boats is an open challenge that needs to be addressed with accessible, scalable, and easy install and use solutions. Despite being based on an algorithm whose performances could be overcome nowadays by more complicated and computational massive neural-network-based algorithms, PortWeather can be considered as a reliable and innovative IOT solution for the real-time weather forecast on little and medium-size boats.

Although the system performs relatively well, there is room for improvement. We plan to install and test new microcontrollers that offer more CPU power as more memory. This will allow us to implement more advanced machine learning algorithms that could help in mitigating the time-lag effect introduced by the simplified regressive algorithm used in PortWeather. On the algorithm performance evaluation, it would be of extreme interest to evaluate the system in heavy weather conditions where the wind direction and speed parameter range are very high. Moreover, we plan to work on the system cloud connection going beyond the pure remote data storage for further analysis. On this side, it could be of extreme interest to allow the system to download historical weather data from publicly available Application Programming Interfaces (APIs) like Weather Underground [59] so that the system will be able to start instantly without the need for the initial training that is actually required at the boot.

## Figures and Tables

**Table 1:** Weather station basic characteristics.

Airmar 220WX	
Operating voltage	Waterproof.
range of 12–24 DC.	
Meteorological Parameters:	Three-axis solid-state
Air Temperature, True/Apparent Wind	compass with dynamic
Speed, True/Apparent Wind Direction,	stabilization (better than 1${}^{\circ }$
Barometric Pressure.	static compass accuracy).
Three-axis accelerometer	Three-axis rate gyros provide
for pitch and roll.	rate-of-turn data.
Internal GPS.	Current Time.

**Table 2:** Description of the provided meteorological parameters.

Measurement	Unit of Measure	Typical Operation Range
**True Wind Speed**	m/s	[0, 60]
**True Wind Direction**	°deg	[0, 360]
**Air Temperature**	°C	[−30, +60]
**Barometric Pressure**	mbar	[540, 1100]
**Relative humidity**	RH%	[0, 100]

**Table 3:** 4ZeroBox specifications.

Power Supply	
Voltage	8 to 36 Vdc
Power Consumption	Typical: 1 W; Maximum: 5 W
**Inputs/Outputs**	
4–20 mA Channels (×4)	Min supported input current 4 mA
(according to switches position)	Max supported input current 20 mA
0–10 V Channels (×4)	Min supported input voltage 0 V
(according to switches position)	Max supported input voltage 10 V
Resistive Channels (×4)	Min supported Resistor value 0 Ω
	Max supported Resistor value 70 KΩ
Current Channels (×3)	Min supported input current −50 mA
	Max supported input current 50 mA
Opto-Isolator Inputs	Input Voltage 5 to 24 V
Relays	10 A–250 V AC (general use) at 40 °C
	8 A–30 V DC (resistive load) at 40 °C
Sinks	60 A–30 V (general use) at 40 °C
Digital I/O	Max supported voltage 3.3 V
**Connections**	
Power Supply, Sensors, RS485,	Pluggable Screw
RS232,CAN, Relays,	Connectors 5.08 pitch
Opto-Isolators, Sinks	
Ethernet	RJ45 Connector
Programming	Micro USB Connector
Li-Po Battery	JST Connector
Micro SD	Micro SD Slot
MikroBus Click Add-on	MikroBus Slots

**Table 4:** Esp32 specifications.

Microcontroller	Tensilica 32-Bit Single-/Dual-Core
	CPU Xtensa LX6
Operating Voltage	3.3 V
Power Supply Voltage:	7–12 V
Digital I/O Pins (DIO)	28
Analog Input Pins (ADC)	8
UARTs	3
SPIs	2
I2Cs	3
Flash Memory	4 MB
SRAM	520 KB
Wi-Fi and Bluetooth	IEEE 802.11 b/g/n/e/i

**Table 5:** Description of the Livorno station dataset.

Measurement	Unit of Measure	Range
**True Wind Speed**	m/s	[1, 19]
**True Wind Direction**	deg	[46, 257]
**Air Temperature**	°C	[+16, +21]
**Barometric Pressure**	mbar	[988, 1003]
**Relative humidity**	%	[72, 95]

**Table 6:** Description of the Pianosa station dataset.

Measurement	Unit of Measure	Range
**True Wind Speed**	m/s	[0, 18]
**True Wind Direction**	deg	[140, 346]
**Air Temperature**	°C	[+16, +20]
**Barometric Pressure**	mbar	[991, 1006]
**Relative humidity**	%	[80, 92]

**Table 7:** Comparing the wind speed prediction error for certain time scale for Livorno weather station.

Time Scale Prediction	MAE	RMSE	MAPE (%)
**30 min**	0.970	1.244	19.34
**60 min**	1.040	1.378	21.03
**90 min**	1.225	1.546	24.70
**120 min**	1.342	1.694	26.77

**Table 8:** Percentage of residual values for Wind Speed prediction for Livorno weather station.

Time Scale Prediction	[−1, 1]	[−2, 2]	[−5, 5]
**30 min**	60%	91%	100%
**60 min**	59%	88%	99%
**90 min**	50%	81%	99%
**120 min**	47%	76%	100%

**Table 9:** Evaluations for wind direction prediction for Livorno weather station dataset.

Time Scale Prediction	MAE	RMSE	MAPE (%)
**30 min**	10.69	15.00	5.84
**60 min**	14.08	18.68	7.62
**90 min**	16.36	21.57	8.77
**120 min**	17.91	23.89	9.61

**Table 10:** Percentage of residual values for Wind Direction for the Livorno Weather Station.

Time Scale Prediction	[−10, 10]	[−15, 15]	[−18, 18]	[−25, 25]
**30 min**	61%	76%	81%	90%
**60 min**	41%	62%	73%	86%
**90 min**	38%	57%	68%	80%
**120 min**	35%	53%	65%	76%

**Table 11:** Comparing the wind speed prediction error for certain time scale for Pianosa weather station.

Time Scale Prediction	MAE	RMSE	MAPE (%)
**30 min**	1.458	2.037	43.48
**60 min**	1.560	2.231	35.65
**90 min**	1.592	2.339	36.12
**120 min**	1.747	2.596	45.22

**Table 12:** Percentage of residual values Wind Speed prediction for the Pianosa weather station.

Time Scale Prediction	[−1, 1]	[−2, 2]	[−5, 5]
**30 min**	47%	74%	97%
**60 min**	48%	72%	95%
**90 min**	47%	71%	95%
**120 min**	43%	70%	94%

**Table 13:** Evaluation for wind direction prediction for the Pianosa weather stations data set.

Prediction	MAE	RMSE	MAPE (%)
**30 min**	16.42	22.25	7.18
**60 min**	17.64	25.75	7.46
**90 min**	20.48	29.16	8.66
**120 min**	22.75	32.50	9.58

**Table 14:** Percentage of residual values—Wind Direction.

Prediction	[−10, 10]	[−15, 15]	[−18, 18]	[−25, 25]
**30 min**	36%	54%	63%	80%
**60 min**	39%	53%	61%	79%
**90 min**	36%	49%	55%	69%
**120 min**	31%	45%	50%	66%

**Table 15:** Description of the dataset from the on boat weather station (Airmar 220WX).

Measurement	Unit of Measure	Range
**True Wind Speed**	m/s	[0, 21]
**True Wind Direction**	deg	[0, 360]
**Air Temperature**	°C	[+4, +19]
**Barometric Pressure**	mbar	[996, 1030]
**Relative humidity**	%	[31, 75]

**Table 16:** Comparing the wind speed prediction error for certain time scale for on boat weather station.

Prediction	MAE	RMSE	MAPE (%)
**30 min**	1.120	1.562	14.26
**60 min**	1.434	1.914	18.18
**90 min**	1.701	2.163	20.12
**120 min**	1.887	2.388	20.64

**Table 17:** Percentage of residual values for the Wind Speed for on boat weather station.

Time Scale Prediction	[−1, 1]	[−2, 2]	[−5, 5]
**30 min**	59%	82%	98%
**60 min**	45%	75%	97%
**90 min**	36%	66%	97%
**120 min**	33%	60%	96%

**Table 18:** Evaluations for wind direction prediction for on boat weather station data.

Time Scale Prediction	MAE	RMSE	MAPE (%)
**30 min**	13.652	30.598	13.81
**60 min**	17.012	34.310	17.44
**90 min**	19.026	36.210	19.26
**120 min**	20.320	36.668	20.93

**Table 19:** Percentage of residual values for Wind Direction for the onboard weather station.

Time Scale Prediction	[−10, 10]	[−15, 15]	[−21, 21]	[−30, 30]
**30 min**	66%	78%	87%	92%
**60 min**	52%	68%	80%	89%
**90 min**	50%	63%	75%	84%
**120 min**	47%	60%	71%	83%

**Table 20:** Benchmark results between the three different stations for the wind speed.

	Livorno			Pianosa			On Boat			Mean		
Time	MAE	MAPE (%)	RMSE	MAE	MAPE (%)	RMSE	MAE	MAPE (%)	RMSE	MAE	MAPE (%)	RMSE
30	0.97	19.34	1.24	1.45	43.48	2.03	1.12	14.26	1.56	1.18	25.69	1.61
60	1.04	21.03	1.37	1.56	35.65	2.23	1.43	18.18	1.914	1.34	24.95	1.84
90	1.22	24.70	1.54	1.59	36.12	2.33	1.70	20.12	2.163	1.50	26.98	2.01
120	1.34	26.77	1.69	1.74	45.22	2.59	1.88	20.64	2.38	1.65	30.87	2.22
**Mean**	1.14	22.96	1.47	1.59	40.11	2.3	1.54	18.30	2.01	1.42	27.12	1.92

**Table 21:** Benchmark results between the three different stations for the wind direction.

	Livorno			Pianosa			On Boat			Mean		
Time	MAE	MAPE	RMSE	MAE	MAPE	RMSE	MAE	MAPE	RMSE	MAE	MAPE	RMSE
30	10.69	5.84	15	16.42	7.18	22.25	13.65	13.81	30.59	13.58	8.94	22.61
60	14.08	18.68	18.68	17.64	7.46	25.75	17.01	17.44	34.31	16.24	14.52	26.24
90	16.36	21.57	21.57	20.48	8.66	29.16	19.02	19.26	36.21	18.62	16.49	28.98
120	17.91	23.89	23.89	22.75	9.58	32.5	20.32	20.93	36.66	20.32	18.13	31.01
**Mean**	14.76	17.49	19.79	19.32	8.22	27.42	17.5	17.86	34.45	17.19	14.51	27.22
